# Development of whole‐genome prediction models to increase the rate of genetic gain in intermediate wheatgrass (*Thinopyrum intermedium*) breeding

**DOI:** 10.1002/tpg2.20089

**Published:** 2021-04-26

**Authors:** Jared Crain, Atena Haghighattalab, Lee DeHaan, Jesse Poland

**Affiliations:** ^1^ Dep. of Plant Pathology Kansas State Univ. 4024 Throckmorton Plant Sciences Center Manhattan KS 66506 USA; ^2^ Stakman–Borlaug Center for Sustainable Plant Health, Center for Applied Phenomics, Univ. of Minnesota 1519 Gortner Avenue St. Paul MN 55108 USA; ^3^ The Land Institute 2440 E. Water Well Rd Salina KS 67401 USA; ^4^ Wheat Genetics Resource Center, Dep. of Plant Pathology Kansas State Univ. 4024 Throckmorton Plant Sciences Center Manhattan KS 66506 USA

## Abstract

The development of perennial grain crops is driven by the vision of simultaneous food production and enhanced ecosystem services. Typically, perennial crops like intermediate wheatgrass (IWG)[*Thinopyrum intermedium* (Host) Barkworth & D.R Dewey] have low seed yield and other detrimental traits. Next‐generation sequencing has made genomic selection (GS) a tractable and viable breeding method. To investigate how an IWG breeding program may use GS, we evaluated 3,658 genets over 2 yr for 46 traits to build a training population. Six statistical models were used to evaluate the non‐replicated data, and a model using autoregressive order 1 (AR1) spatial correction for rows and columns combined with the genomic relationship matrix provided the highest estimates of heritability. Genomic selection models were built from 18,357 single nucleotide polymorphism markers via genotyping‐by‐sequencing, and a 20‐fold cross‐validation showed high predictive ability for all traits (*r > *.80). Predictive abilities improved with increased training population size and marker numbers, even with larger amounts of missing data per marker. On the basis of these results, we propose a GS breeding method that is capable of completing one cycle per year compared with a minimum of 2 yr per cycle with phenotypic selection. We estimate that this breeding approach can increase the rate of genetic gain up to 2.6× above phenotypic selection for spike yield in IWG, allowing GS to enable rapid domestication and improvement of this crop. These breeding methods should be transferable to other species with similar long breeding cycles or limited capacity for replicated observations.

## Core Ideas

-  Adding molecular markers in phenotypic analysis increased heritability estimates.
-  Genomic selection (GS) resulted in high accuracy in a wheatgrass breeding program.
-  GS predictions improved with increasing marker number and training population size.
-  Up to a 2.6‐fold increase in genetic gain for spike yield could be achieved with GS.
-  GS reduced cycle time by half, potentially doubling genetic gains.

**Abbreviations**
AIC: Akaike information criterion
AR1: autoregressive first order
BLUP: best linear unbiased prediction
GBLUP: genomic best linear unbiased prediction
GBS: genotyping‐by‐sequencing
GEBV: genomic estimated breeding value
GRM: genomic relationship matrix
GS: genomic selection
iid: independently and identically distributed
IWG: intermediate wheatgrass
PS: phenotypic selection
SNP: single nucleotide polymorphism
TLI: The Land Institute.

## 1 INTRODUCTION

Perennial crops can provide a host of ecosystem services and increase sustainability (Jordan et al., 2007; Rasche et al., 2017). The environmental benefits realized by perennial crops include reduced nitrate leaching and increased water utilization (Culman et al., 2013), increased soil organic carbon (Culman et al., 2010), and greater species diversity above and below ground, allowing for more robust food webs (Culman et al., 2010; Glover et al., 2010a). Even though perennial crops provide many unparalleled services compared with annual cropping systems, they are grown on less than 40% of the world's arable land (Cox, Glover, Van Tassel, Cox, & DeHaan, 2006; Glover, Cox, & Reganold, 2007). Given the potential benefits of perennial crops, there is a strong interest in increasing the use of these plants. However, although the benefits of perennial crops are evident, currently, all the major cereal crops are annuals. Work to produce new perennial grain crops began as early as 1930s (Armstrong, 1936; Kane, Rogé, & Snapp, 2016) but have been challenged by complex biology, long lifecycles, undomesticated species as the starting germplasm for perennial grain crops, and limited resources and technology (Cox et al., 2002; Glover et al., 2010b).

During a search for new crops for sustainability, intermediate wheatgrass (IWG)[*Thinopyrum intermedium*(Host) Barkworth & D.R Dewey] was identified by the Rodale Institute (Kutztown, PA, USA) for its relatively large seed size and potential as a perennial grain crop (Wagoner, 1990a; 1990b). Intermediate wheatgrass was first brought to the United States for erosion control in the 1930s from Russia (Vogel & Jensen, 2001). It has high biomass yield (Harmoney, 2015) and can provide ecosystem services (Culman et al., 2010; Culman, Snapp, Ollenburger, Basso, & DeHaan, 2013; Glover, 2010), as well as being used as a source of grain for human consumption. Quality evaluation has shown IWG has superior amino acid levels, higher protein, and higher bran percentage than wheat (*Triticum aestivum* L.) (Becker, Wagoner, Hanners, & Saunders, 1991). Further research has demonstrated that 50:50 mixtures of IWG flour and wheat increased nutritional properties while retaining sufficient baking quality (Marti, Qiu, Schoenfuss, & Seetharaman, 2015).

**FIGURE 1:**
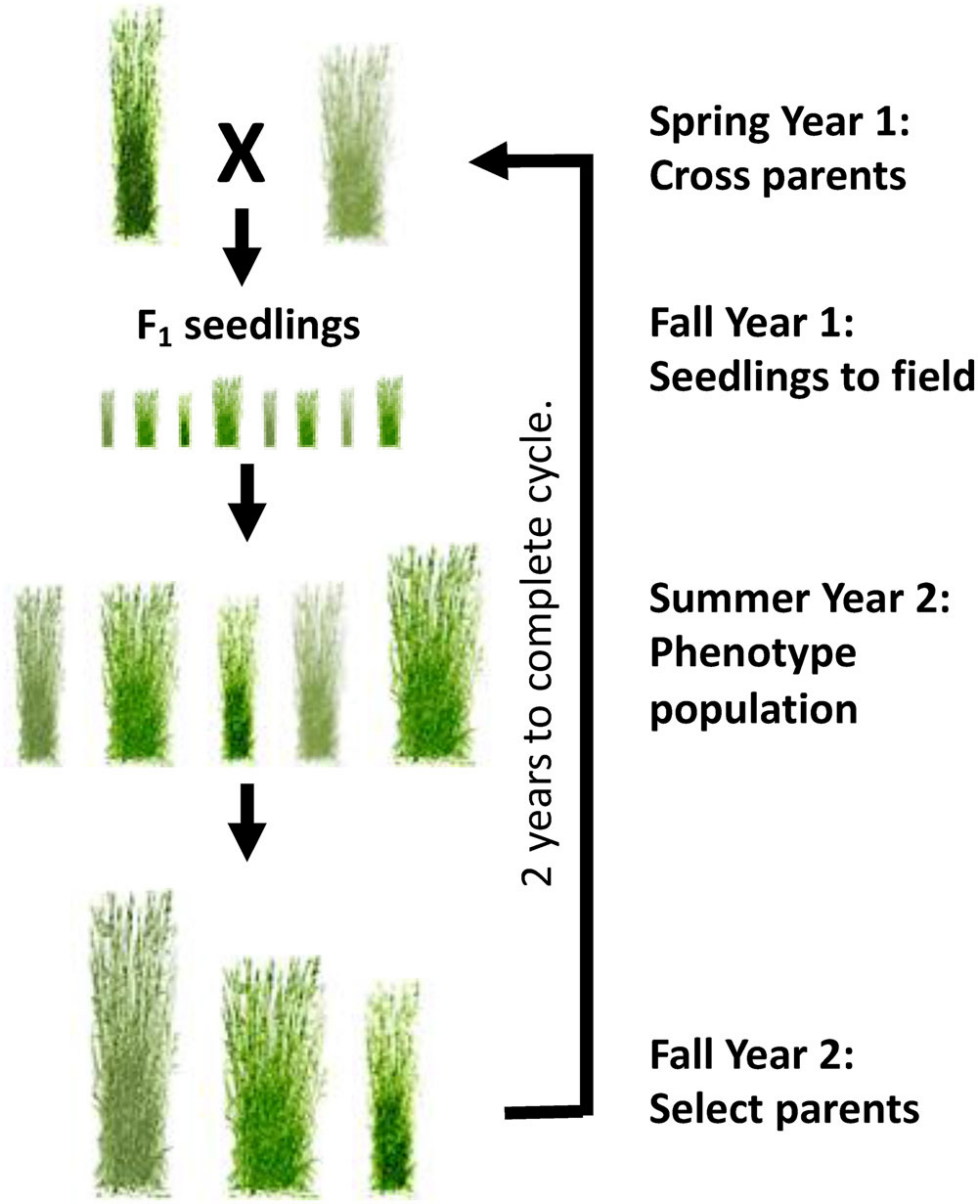
Diagram of the intermediate wheatgrass phenotypic selection breeding program used by The Land Institute, Salina, KS

In 1988, a breeding initiative was started at the Rodale Institute to domesticate IWG, and two cycles of selection were performed at the Big Flats Plant Materials Center (Corning, NY) to increase seed size and fertility (DeHaan et al., 2014). Beginning in 2001, The Land Institute (TLI) (Salina, KS) started a breeding program using selections from the USDA's Big Flats Plant Materials Center (DeHaan, Christians, Crain, & Poland, 2018). Intermediate wheatgrass is a highly diverse and heterozygous species, and the breeding program is largely defined by pedigree‐based selection followed by controlled crossing of selected parents. The IWG breeding program has consistently undergone changes as it has developed and matured, as outlined by DeHaan et al. (2018). In general, the IWG program has been based on phenotypic selection (PS) and it currently takes, at minimum, 2 yr to complete one cycle of selection (Figure 1). Phenotypic selection has been successful highlighted by the fact that seed yield increased by 77% after two cycles of selection. However, to match the productivity of annual wheat yield, it was projected to take another 20 yr of selection at the same level of genetic gains (DeHaan et al., 2014). For seed size, DeHaan et al. (2014) suggested that it would take over 100 yr to reach the same size as annual wheat, and it is clear that greater genetic gains are required to develop IWG into a mainstream crop.

**Core Ideas**

-  Adding molecular markers in phenotypic analysis increased heritability estimates.
-  Genomic selection (GS) resulted in high accuracy in a wheatgrass breeding program.
-  GS predictions improved with increasing marker number and training population size.
-  Up to a 2.6‐fold increase in genetic gain for spike yield could be achieved with GS.
-  GS reduced cycle time by half, potentially doubling genetic gains.

Given the magnitude of the progress required, new technology and breeding methodologies are needed to accelerate IWG breeding. Possible strategies to increase the rate of genetic gain are through application of genomic technologies (Tester & Langridge, 2010) and enhanced phenotypic evaluation. Nearly all advances in IWG have been made through PS, as genetic technology and markers have historically been time‐ and cost‐prohibitive (Davey et al., 2011; Xu & Crouch, 2008). However, advances in next‐generation sequencing technology have provided breakthroughs to study challenging species like IWG that have large genome size and complexity from polyploidy (12.6 Gb, allohexaploid 2*n* = 6*x* = 42). In a period of 5 yr, IWG was taken from a species with virtually no genomic resources to having genetic maps and a drafted reference genome (Kantarski et al., 2016; Larson et al., 2019; Zhang et al., 2016).

Using genotyping‐by‐sequencing (GBS)(Elshire et al., 2011; Poland, Brown, Sorrelss, & Jannink, 2012), Kantarski et al. (2016) developed the first consensus genetic map of IWG from 13 heterozygous parents. Continuing with molecular advances, Zhang et al. (2016) published the first account of genomic selection (GS) in IWG. They predicted that coupling the development of genetic maps with the power of next‐generation marker discovery and genomic profiling, genomic technology should increase the rate of genetic gain in IWG. Zhang et al. (2016) showed that GS could enhance breeding efficiency compared with phenotypic recurrent selection alone, and they proposed a method to implement GS in an IWG breeding program. Although they suggested implementing GS alongside PS, this breeding method still required 2 yr per cycle, which would not take advantage of large cycle time reductions, one of the biggest potential benefits of GS (Heffner, Lorenz, Jannink, & Sorrells, 2010).

In addition to new molecular tools and techniques, new strategies have been suggested to accurately phenotype IWG in the field. Because IWG is a heterozygous plant and replication is only possible through cloning, IWG is often evaluated as single entries with no replication, limiting the use of powerful replicated field experiments. To address the unreplicated field designs, the TLI breeding program has routinely used row–column autoregressive order 1 (AR1) × AR1 models (Gilmour, Cullis, & Verbyla, 1997). However, many other models to handle spatially dependent data have been postulated, and the analysis of field experiments to obtain the best assessment of genetic or experimental value has a long history. One of the earliest references to adjusting values based on spatial location is Papadakis (1937). On the basis of these methods, nearest‐neighbor analysis has evolved to adjust plot values by the surrounding neighborhood (local) value (Bartlett, 1978; Peiris, Samita, & Veronica, 2008; Piepho, Richter, & Williams, 2008; Wilkinson, Eckert, Hancock, & Mayo, 1983). Work by Lado et al. (2013) showed that use of a moving grid covariate resulted in more accurate phenotypes and higher GS prediction accuracies for wheat. Further work has relaxed the assumption of adjustment by rows and columns and can use spatial distance to create a moving circular grid of varying sizes (Haghighattalab et al., 2017). Improved models to account for variable field conditions would further improve both the development of accurate GS prediction models and the accuracy of PS per se.

We evaluated how to optimally transition an IWG breeding program from PS to GS. Our objectives were to (a) determine optimal methods to obtain genotypic values from field trials by reducing spatial variation, (b) evaluate the potential of GS to predict traits and how best to leverage genomic data within the breeding program, and (c) estimate potential genetic gains from applying GS in an integrated pipeline strategy for IWG breeding.

## 2 MATERIALS AND METHODS

### 2.1 The Land Institute‐Cycle 6 population

Our study used the sixth cycle of selection (TLI‐Cycle 6) at TLI (Salina, KS). Using terminology that is consistent with Zhang et al. (2016), we use the term genet to refer to a genetically unique entity with its own genetic makeup. Genets are typically represented by a single plant early in the breeding cycle, but they can be cloned to form multiple individual plants (ramets) derived from the same genet. TLI‐Cycle 6 comprised 3,658 genets that were the progeny of 109 unique parents from the TLI‐Cycle 5 generation. All TLI‐Cycle 6 genets were derived from pairwise crosses between TLI‐Cycle 5 genets, resulting in 674 unique full‐sib families (1–10 plants per family). DeHaan et al. (2018) provided further details about developing the TLI‐Cycle 6 population and information about how the parents of the TLI‐Cycle 6 generation were selected within the breeding program.

Genets were started in the greenhouse in 4.5‐ by 4.5‐ by 5.5‐cm pots (Jiffy Products of America). Once the genets were established, they were tissue‐sampled for DNA and moved to the field (38.7690° N, 97.5954° W) in the fall of 2015 and placed on a grid with 61 cm between genets and a row spacing of 91.4 cm. Genets were completely randomized, with the relative field position of each individual recorded by a Trimble real‐time kinematic positioning system with 1‐cm accuracy. Each genet was only replicated once and there were no control replicates because of the logistical challenges of cloning check genets and developing historical knowledge about the check's performance. Phenotypic data were collected for 2 yr during 2016 and 2017 for a variety of traits. Although 46 traits were measured over the 2 yr, the traits of greatest interest to the breeding program, referred to as priority traits, were percentage of free threshing, reduced shattering, spike yield, and seed mass. Percentage of free threshing is a visual estimate of 0 to 100% of seed free of the lemma and palea after threshing. Shattering was evaluated prior to threshing and rated on a 0 to 4 scale, where 0 indicates that the spikelets could be bent 90° relative to the rachis without breaking, 1 indicates that no florets broke off the rachis after dropping from a height of 25 cm three times, 2 has one to five florets shattering after dropping, 3 has more than five florets shattering after dropping, and 4 indicates that more than 50% of the florets shattered (DeHaan et al., 2018). Spike yield was measured by threshing 5 to 10 of the largest mature heads from each genet, with seed mass being calculated from about 20 undamaged kernels per genet (DeHaan et al., 2018). All traits, the number of observations, and the sample statistics are compiled in Supplemental Table S1. Because of flooding and time constraints, not all traits were collected across all genets, creating a dataset that had a significant amount of missing data.

### 2.2 Statistical analysis of phenotypic data

All statistical analysis was completed in the R programming language (R Core Team, 2017). Several phenotypic models were used to evaluate how different models would affect the GS results and assessments of genet performance. There were 77 genets that were known to be from self‐pollinations during crosses, and these were removed to avoid biasing GS prediction results or heritability calculations.

In general, two types of models were assessed: models that used a residual structure variance–covariance matrix to model the spatial dependency throughout the field, and models that used a spatial covariate (e.g., moving means covariate) similar to Lado et al. (2013) to adjust the phenotypic value based on the local location.

The first set of models was based on the mixed linear model and had the general form of (Isik, Holland, & Maltecca, 2017):

(1) y=Xb+Zu+e,

ASREML‐R (Butler, 2009) was used to fit the mixed model, where ***y*** is a vector of phenotypic observations; ***X*** and ***Z*** are incidence matrices for fixed components and random components, respectively; ***b*** and ***u*** are vectors of fixed and random effects; and ***e*** is a vector of random residual effects. The phenotypic vector ***y*** is distributed as **y** ∼ N(**Xb**, **V**), where ***y*** is normally distributed with a mean of ***Xb*** and a variance of ***V***. The total variance is $V\;=\;\;\left(\begin{array}{c} u\\ e \end{array}\right)=\;\left(\begin{array}{cc} G & 0\\ 0 & R \end{array}\right)$, where **G** is the genetic variance component normally distributed as $G\;∼N(0,\sigma_{u}^{2}A)$ with a mean of 0, and a variance–covariance as genotypic variance ($\sigma_{u}^{2})$ times the additive genetic relationship (**A** matrix) from the pedigree.

The **R** structure in this model accounts for the spatial effects, with **R** fitting a row–column AR1 model described as:

(2) $$R\;=\;\sigma_{e}^{2}\Sigma_{c}\left(\rho_{c}\right)\;⊗\;\Sigma_{r}\left(\rho_{r}\right)\;,$$

where $\sigma_{e}^{2}$ is the residual error variance, ρ is the correlation parameter for the AR1 model, $\Sigma_{c}\left(\rho_{c}\right)$ ****is a matrix of AR1 column correlations equal to the number of columns, and $\Sigma_{r}\left(\rho_{r}\right)$ is a matrix of AR1 row correlations equal to the number of rows. The Kronecker product (⊗) expands the rows by columns to equal the total number of rows by columns in the experimental site. ASREML requires the experimental design for AR1 models to be rectangular (complete observations for all rows by columns), so dummy variables were added to rows and columns to get a complete rectangle of 16 rows by 248 columns (3,968 total entries).

Other random effects, such as parent interactions, were included by adding random terms as $\;∼N(0,\sigma_{\left(r_{t}\right)}^{2}I)$, where the random term is distributed normally, with a mean of 0, and a variance of $\sigma_{\left(r_{t}\right)}^{2}$. By Equation 1, several models were developed to account for spatial effects within the mixed model framework. For consistency, all output from these models will be referred to as best linear unbiased predictions (BLUPs).

The first model fits only field effects and main effects for maternal and paternal parents and does not use genetic relationships from pedigree or markers, but only a random effect for genets to estimate genet effect and variance components (Jordan, Potts, & Wiltshire, 1999).

(3) $$\mathrm{Model}\;1\;\;:\;y_{ijk}=\mu +g_{i}+m_{j}+\;f_{k}+\;mf_{\mathrm{jk}}+\;e_{\mathrm{ijk}},$$

where $y_{ijk}$ is the observed phenotype for genet *i* (*g_i_*), with the male parent *j* (*m_j_*), and the female parent *k* (*f_k_*), which is independently and identically distributed (iid) as $y_{ijk}$ ∼ N(**Xb**, **V**). Random terms forming the variance (**V**) include a genet effect iid as $g_{i}$ ∼ N(0, $\sigma_{g}^{2}$), the male parent iid as $m_{j}$ ∼ N(0, $\sigma_{m}^{2}$), the female parent effect iid as $f_{k}$ ∼ N(0, $\sigma_{f}^{2}$), and the parent interaction effect iid distributed $mf_{jk}$ ∼ N(0, $\sigma_{mf}^{2}$). The correlated residual, $e_{ijk}$, accounts for the spatial effects in the mixed model in the **R** structure with an AR1 × AR1 (row, column) design.

The second and third models are similar to Model 1 with the addition of genetic effects estimated from pedigree (Model 2) and from markers (Model 3). Model 2 with pedigree is designated as follows:

(4) $$\begin{array}{ccc} \mathrm{Model}\;2\;\;:\;y_{ijk} & = & \mu +\mathrm{gen}o_{i}+m_{j}+f_{k}+\;mf_{\mathrm{jk}}+\mathrm{units}\\ & & +\;e_{ijk}, \end{array}$$

The terms are similar to those in Model 1, for the observations of interest, population mean, male, female, parent interaction, and residual structure. The genet term has been replaced by geno, where the genetic relationship is modeled by pedigree as the additive relationship matrix (**A**), with geno distributed as N(0, **A**). The units term (measurement error) is iid as ∼ N(0, σ^2^), and it separates residual variance into an independent residual structure and a nonindependent residual structure attributable to the correlated (AR1 × AR1) model terms (Isik et al., 2017). This has been the typical model used in previous generations to evaluate phenotypic traits (DeHaan et al., 2018). The pedigree for phenotypic correction has often been used in animal breeding, where each observation unit has its own genetic makeup, very similar to IWG (Isik et al., 2017; Lynch & Walsh, 1998; Mrode, 2005).

(5) $$\begin{array}{ccc} \mathrm{Model}\;3\;\;:\;y_{ijk} & = & \mu +\mathrm{gen}o_{i}+m_{j}+\;f_{k}+\;mf_{\mathrm{jk}}+\mathrm{units}\\ & & +\;e_{ijk}. \end{array}$$

This is equivalent to Model 2, but the **A** matrix has been replaced by the realized genomic relationship matrix (GRM) (Isik et al., 2017) calculated from single nucleotide polymorphism (SNP) markers using the *rrBLUP* package (Endelman, 2011) with geno distributed as N(0, **GRM**).

(6) $$\begin{array}{ccc} \mathrm{Models}\;4\;\mathrm{to}\;6\;\;:\;y_{ijk} & = & \mu +\beta_{i}+\mathrm{gen}o_{i}+m_{j}+\;f_{k}+\;mf_{\mathrm{jk}}\\ & & +\;e_{ijk}. \end{array}$$

Here, the model terms are equivalent to those in Model 3, with the exception of $\beta_{i}$ and $e_{ijk}$. A covariate term, $\beta_{i}$, which represents the mean moving average adjustment at varying sizes (1.5, 3, and 6 m) from the genet of interest, has been added to complete spatial correction. The AR1 × AR1 residual structure has been dropped from the model with $e_{ijk}$ iid as N(0, $\sigma_{e}^{2}$).

Each model was computed for all traits, and heritability and the Akaike information criterion (AIC)(Akaike, 1974) were used as an indicators of model fit. For all models, AIC differences between models were compared according to Burnham and Anderson (2002).

Narrow‐sense heritability (*h* ^2^) was calculated as (Jordan et al., 1999):

(7) $$h^{2}=\;\frac{\sigma_{additive}^{2}}{\sigma_{phenotypic}^{2}},$$

where:

(8) $$\begin{array}{ccc} \sigma_{phenotypic}^{2} & = & \;\sigma_{additive}^{2}+\;\sigma_{male}^{2}+\;\sigma_{female}^{2}+\;\sigma_{interaction}^{2}\\ & + & \;\sigma_{error}^{2}, \end{array}$$

and $\sigma_{phenotypic}^{2}$ is the total phenotypic variance; $\sigma_{additive}^{2}$ is additive genetic variance; $\sigma_{male}^{2},\;\sigma_{female}^{2},\;\mathrm{and}\;\sigma_{interaction}^{2}$ are the paternal, maternal, and nonadditive variances, respectively; and $\sigma_{error}^{2}$ is the residual error variance. For Models 1 to 3, the residual error variance was the nugget effect after fitting the AR1 × AR1 residual structure (Butler, Cullis, Gogel, & Thompson, 2017). In Models 4 to 6, the error variance was the residual error. Standard errors of the heritability methods were computed via the Delta method (Isik et al., 2017).

### 2.3 Genomic profiling

Genotyping‐by‐sequencing was used to profile 3,037 of the TLI‐Cycle 6 genets with two restriction enzymes (*PstI* and *MspI*) following the methods of Poland et al. (2012). The TASSEL GBSv2 pipeline was used to call SNPs (Glaubitz et al., 2014), with the draft IWG reference genome (access provided by The *Thinopyrum intermedium* Genome Sequencing Consortium, https://phytozome‐next.jgi.doe.gov/info/Tintermedium_v2_1, accessed 16 Mar. 2021). The initial genotype calling identified 131,879 putative SNPs.

The genetic data were filtered to eliminate samples and loci with low reads. The filtering proceeded as follows: (a) tags that aligned to multiple locations in the genome were discarded; (b) only biallelic SNP markers were used; (c) each homozygous genotype had to have a minimum of four tag counts (sequence depth) per locus per individual (If this threshold was not met, the call was set to missing. For heterozygotes, a minimum of two tags representing the contrasting alleles were required to call the locus genotype.); (d) SNPs were filtered to have a minor allele frequency greater than 0.01; (e) each SNP locus could have no more than 70% missing data. After filtering for locus quality, individuals that had more than 95% missing data were discarded. The final dataset after filtering consisted of 3,011 genets and 18,357 SNP markers. Missing data were imputed by Beagle version 4.1 (Browning & Browning, 2016).

### 2.4 Genomic selection models

To determine if traits could be predicted from molecular markers, several GS models were evaluated. For each trait, we used a 20‐fold cross‐validation, where 95% of the genets with observations formed the training population and the other 5% of the genet observations were masked, forming the prediction population, with several common GS models including genomic best linear unbiased prediction (GBLUP), Gaussian kernel, BayesA, BayesB, BayesCπ, LASSO, and GBLUP model with dominance. The output of the GS models is referred to as genomic estimated breeding values (GEBVs).

To evaluate the models, the correlation between the GS‐predicted GEBVs and the BLUPs were considered as the GS predictive ability, with the confidence intervals being computed by the *psychometric* package (Fletcher, 2010). The GBLUP and Gaussian kernel models were fitted by the *rrBLUP* package (Endelman, 2011), whereas other models were fitted by the *BGLR* package (Pérez & De Los Campos, 2014). To fit the GBLUP model with dominance, a dominance relationship matrix was made by the *sommer* package (Covarrubias‐Pazaran, 2016), and both the GRM and dominance relationship matrix were passed to the BGLR function as a multi‐kernel model (Momen & Morota, 2018). Bayesian models were fitted with a burn‐in of 15,000, then 35,000 iterations.

### 2.5 Factors effecting GS accuracy

With the large dataset (∼2,200 observations per trait, 18,357 markers), we also investigated how the composition of the training set, marker number, and training population size would affect the GS predictive ability. We evaluated the effect of several k‐fold cross‐validation designs, where the levels of cross‐validation were completed by randomly partitioning the dataset into training and prediction sets (4:1, 9:1, 14:1, and 19:1). To generate predictions for all individuals, the model was recalculated for each prediction set with all remaining partitions in the training set, so for the 20‐fold cross‐validation, one set of data was predicted by the other 19 data sets as the training population, followed by refitting the model for each of the 20 partitions until all genets had been predicted. With 100 iterations of random sampling, this changed the size (*k*‐fold validations) and the genets that were in the training population. Predictive ability was determined by calculating the correlation between the BLUPs and the GEBVs from the GS model.

As the marker data could contain a significant amount of missing data before imputation, we also evaluated how the percentage present (number of SNP sites per locus called on all individuals) effected GS predictive ability. With the same 20‐fold cross‐validation, predictive ability was determined by varying levels (250–17,500) of randomly drawn marker sets and marker sets selected by the percentile of percentage of present calls. For randomly drawn markers, the process was repeated for 100 iterations for each level.

Along with marker number, we also investigated how the size and composition of the individuals in the training population would affect predictive ability. Training population size was evaluated for levels varying from 250 to 2,000 (or the maximum number of individuals) with all markers. Similar to the effect of marker number, random samples for each size of the training population were drawn, followed by predicting all remaining individuals that were not used to train the model. Accuracy was determined for each of the 100 iterations as the correlation between BLUPs and the GEBVs. In addition to randomly selecting the training population, training populations were also based on the family relationships, which should prevent closely related individuals from upwardly biasing predictions. This method was similar to that of Zhang et al. (2016), where training populations were specifically comprised and validated by masking half‐sib families. One half‐sib family was selected as the prediction set with the common parent excluded (either the father or mother) from the training set. The training population then comprised the remaining half‐sib families with individuals from each family randomly selected to train the model. Testing was completed with the training population comprising 1 to 20 individuals from each half‐sib family to determine how the number of individuals per family influenced GS predictions. This allowed the GEBVs to be made on each half‐sib family without any direct relationship to the common parent in the training set. For comparisons, if groups had nonoverlapping correlation 95% confidence intervals, we considered them statistically significant.

Finally, on the basis of results of marker number and training population size, we completed 100 iterations for combinations of marker number and training population size. This process was completed similarly to individual comparisons of marker number and training population size, with samples being drawn at random, and a 20‐fold cross‐validation and predictive ability computed as the correlation between BLUPs and the GEBVs.

### 2.6 Potential for genetic gain

In order to estimate the ability of GS to impact the IWG breeding program, we evaluated the potential to increase genetic gain via indirect selection. Indirect selection can be considered as (Falconer & Mackay, 1996):

(9) $$\frac{CR}{R}=\;\frac{i_{CR}h_{CR}r_{A}}{i_{R}h_{R}},$$

where *CR* is the correlated response from indirect selection; *R* is the response of the trait under direct selection; *i* is the selection intensity expressed in units of phenotypic SD for *CR* and *R*, respectively; *h* is the square root of heritability with values for both *CR* and *R*; and *r_A_* is the additive genetic correlation. In the approach of Battenfield et al. (2016), the phenotypic correlation (*r_P_*) between two traits is modeled as (Falconer & Mackay, 1996):

(10) $$r_{P}=\;h_{R}h_{CR}r_{A}+\;e_{R}e_{CR}r_{E},$$

where *e* is the environmental correlation. If we assume that environments are unrelated, Equation 10 reduces to:

(11) $$r_{P}=\;h_{R}h_{CR}r_{A}.$$

Following rearrangement, additive genetic correlation can be expressed as:

(12) $$r_{A}=\;\frac{r_{P}}{h_{R}h_{CR}},$$

After substitution of Equation 12 into Equation 9 and rearrangement, the relative efficiency of indirect selection from GS can be calculated on a cycle basis as:

(13) $$\frac{CR}{R}=\;\frac{i_{CR}r_{P}}{i_{R}h_{R}^{2}}.$$

Equation 13 can be divided by the quotient of years to complete GS and PS cycles to evaluate the relative efficiency (*RE*) for comparing breeding cycles of different lengths of time as:

(14) $$RE\;=\;\frac{\frac{i_{CR}r_{P}}{i_{R}h_{R}^{2}}}{GS/PS}.$$

This formulation is similar to dividing the breeders’ equation by years to complete a cycle for yearly genetic gain (Fehr, 1987) and assumes that genetic parameters, including genetic variance and heritability, remain the same across cycles. To assess a realistic population size and selection intensity within the IWG breeding program limitations, we used the following estimates, given the currently available resources. Direct selection can be assessed on up to 10,000 genets per cycle, with 100 plants selected as parents, resulting in a direct selection intensity of 1% (*i_R_* = 2.665). Under similar expected resources, up to 4,000 genets could be genotyped, again selecting 100 genets as parents for the following cycle, resulting in an indirect selection intensity of 2.5% (*i_CR_* = 2.338). Phenotypic correlation for Equation 13 was the correlation between GS GEBVs in 2016 and the 2017 BLUPs, similar to methods used by Battenfield et al. (2016).

### 2.7 Data analysis and availability

All data analyses were carried out in R (R Core Team, 2017). All genotypic data have been uploaded to the NCBI sequence read archive (https://www.ncbi.nlm.nih.gov/bioproject/, accessed 16 Mar. 2021) as BioProject accession number PRJNA563706. All phenotypic data and scripts for analysis have been made available in the Dryad Digital Repository: https://doi.org/10.5061/dryad.73n5tb2t9 (accessed 16 Mar. 2021).

## 3 RESULTS

### 3.1 Method of spatial correction

Across all traits, Model 3, which includes the GRM and an autoregressive row–column design, consistently provided the best heritability and model fit for the phenotypic data (Table 1). For priority traits, we found moderate heritability ranging from 0.24 to 0.73 for free threshing, 0.14 to 0.38 for shattering, 0.19 to 0.52 for seed mass, and 0.20 to 0.54 for spike yield. Across all models a few trends emerged. The model with the GRM (Model 3) consistently performed better than those with the pedigree relationship matrix (Model 2), indicating that the genomic markers had captured the realized relationships. We did not observe a significant improvement with spatial corrections with moving means covariate adjustment (Models 4–6), as these models were within the estimated SE of the models with residual variance structures (Models 1 or 3), or the AIC differences did not provide compelling evidence to choose the spatial correction of Models 4 to 6 over the next best performing model. Across 46 different traits, Model 3 provided the highest estimated for heritability for 70% of the traits and the lowest AIC for 46% of the traits, supporting the conclusion that this model accounting for maternal and paternal effects along with the GRM and an autoregression row–column model of the residual variance was the most robust model overall for these IWG field trials. On the basis of these findings, all GS results, other than where specifically noted, are from BLUPs from Model 3.

**TABLE 1:** Narrow‐sense heritability (*h* ^2^), estimate of the SE of heritability, and Akaike information criterion (AIC) for intermediate wheatgrass traits evaluated by six different models to account for spatial variation. Data were collected during 2016 and 2017 in Salina, KS

		Model 1			Model 2			Model 3			Model 4			Model 5			Model 6		
Trait	Year	*h* ^2^	SE	AIC	*h* ^2^	SE	AIC	*h* ^2^	SE	AIC	*h* ^2^	SE	AIC	*h* ^2^	SE	AIC	*h* ^2^	SE	AIC
Flag leaf height (cm)	2016	0.28	0.04	9,909	0.25	0.07	9,545	**0.51**	**0.06**	**7,765**	0.45	1.29	7,718	0.49	1.26	7,739	0.48	1.28	7,752
Flag leaf length (cm)	2016	0.19	0.03	11,002	0.23	0.06	10,692	0.32	0.04	8,858	0.33	0.25	8,853	**0.33**	**0.25**	**8,856**	**0.33**	0.25	8,856
Flag leaf width (mm)	2016	0.35	0.04	7,978	0.27	0.06	7,729	0.50	0.04	6,327	**0.50**	**0.11**	**6,307**	0.50	0.11	6,306	0.50	0.11	6,317
Florets per spikelet	2016	0.13	0.03	4,005	0.21	0.06	3,885	0.33	0.05	3,172	**0.33**	**0.07**	**3,190**	0.33	0.07	3,188	0.32	0.07	3,180
Florets per spikelet	2017	0.05	0.02	3,573	0.01	0.02	3,460	**0.11**	**0.03**	**3,419**	0.09	0.03	3,494	0.09	0.03	3,511	0.10	0.03	3,509
**Free threshing %^a^**	2016	**0.53**	**0.04**	**15,578**	0.39	0.08	15,002	0.53	0.05	12,345	0.51	3.88	12,453	0.51	3.87	12,483	0.5	3.99	12,515
**Free threshing %**	2017	**0.41**	**0.04**	**16,140**	0.24	0.06	15,579	0.38	0.04	15,405	0.36	2.57	15,431	0.36	2.56	15,453	0.36	2.53	15,459
Maturity	2016	0.37	0.04	10,430	0.27	0.06	10,067	**0.62**	**0.04**	**8,097**	0.60	0.12	8,123	0.61	0.12	8,099	0.61	0.12	8,118
Number of florets per spike	2016	0.20	0.03	19,452	0.30	0.07	18,886	**0.42**	**0.05**	**15,570**	0.40	19.33	15,540	0.40	19.2	15,590	0.39	19.13	15,582
Number of florets per spike	2017	0.11	0.03	19,810	0.07	0.04	19,282	**0.12**	**0.03**	**19,172**	0.10	5.35	19,257	0.11	5.78	19,298	0.12	6.16	19,302
Percent seed set	2016	0.24	0.04	–7,221	0.25	0.06	–7,080	**0.35**	**0.05**	**–5,837**	0.32	0.05	–5,795	0.33	0.05	–5,830	0.33	0.05	–5,845
Percent seed set	2017	0.15	0.03	–7,522	0.15	0.05	–7,361	**0.21**	**0.04**	**–7,327**	0.18	0.04	–7,171	0.17	0.04	–7,194	0.17	0.04	–7,194
Percent spike emergence	2017	0.27	0.04	–3,415	0.31	0.06	–3,380	**0.46**	**0.04**	**–3,467**	0.42	0.04	–3,426	0.42	0.04	–3,435	0.44	0.04	–3,451
Plant height (cm)	2016	0.31	0.05	11,071	0.24	0.07	10,699	**0.63**	**0.06**	**8,658**	0.58	2.39	8,599	0.58	2.33	8,654	0.58	2.39	8,654
Plant height (cm)	2017	0.17	0.04	14,538	0.15	0.05	14,127	**0.44**	**0.04**	**13,930**	0.39	1.48	13,972	0.40	1.45	13,960	0.40	1.48	13,979
Seed area (mm^2^)	2016	0.52	0.04	2,284	0.37	0.07	2,082	**0.60**	**0.04**	**1,579**	0.56	0.05	1,714	0.59	0.05	1,657	0.57	0.05	1,666
Seed area (mm^2^)	2017	**0.44**	**0.04**	**3,021**	0.29	0.06	2,841	0.37	0.04	2,801	0.32	0.05	3,034	0.31	0.05	3,055	0.31	0.05	3,140
Seed density	2016	0.23	0.04	–6,217	0.13	0.05	–6,111	**0.43**	**0.05**	**–5,155**	0.37	0.05	–5,050	0.39	0.05	–5,115	0.39	0.05	–5,116
Seed density	2017	0.25	0.04	–7,291	0.22	0.06	–7,137	**0.33**	**0.04**	**–7,163**	0.26	0.04	–6,974	0.26	0.04	–6,909	0.25	0.04	–6,767
Seed image circularity	2016	0.44	0.04	–12,657	0.30	0.07	–12,466	**0.63**	**0.04**	**–10,576**	0.62	0.04	–10,524	0.62	0.04	–10,579	0.62	0.04	–10,583
Seed image circularity	2017	0.30	0.04	–13,300	0.19	0.06	–13,010	**0.47**	**0.04**	**–13,094**	0.39	0.04	–12,880	0.43	0.04	–12,940	0.42	0.04	–12,894
Seed length (mm)	2016	0.59	0.04	–993	0.45	0.08	–1,129	**0.70**	**0.04**	**–1,154**	0.63	0.04	–1,006	0.66	0.04	–1,066	0.65	0.04	–1,069
Seed length (mm)	2017	**0.53**	**0.04**	**–763**	0.41	0.07	–859	0.51	0.04	–1,000	0.50	0.04	–893	0.50	0.04	–928	0.50	0.04	–947
**Seed mass (mg)**	2016	0.38	0.04	4,485	0.19	0.06	4,244	**0.50**	**0.05**	**3,422**	0.45	0.07	3,537	0.48	0.07	3,473	0.47	0.07	3,456
**Seed mass (mg)**	2017	**0.41**	**0.04**	**3,902**	0.27	0.06	3,696	0.41	0.04	3,595	0.38	0.05	3,684	0.39	0.05	3,582	0.38	0.05	3,630
Seed perimeter (mm)	2016	0.59	0.04	2,456	0.43	0.08	2,229	**0.70**	**0.04**	**1,639**	0.63	0.05	1,781	0.66	0.05	1,731	0.65	0.05	1,731
Seed perimeter (mm)	2017	**0.52**	**0.04**	**2,989**	0.39	0.07	2,795	0.49	0.04	2,663	0.47	0.05	2,788	0.47	0.05	2,768	0.47	0.05	2,766
Seed width (mm)	2016	0.37	0.04	–7,172	0.26	0.06	–7,088	0.55	0.05	–6,055	0.54	0.05	–5,951	**0.55**	**0.05**	**–6,022**	0.54	0.05	–6,016
Seed width (mm)	2017	**0.29**	**0.04**	**–6795**	0.14	0.05	–6,675	0.27	0.04	–6,656	0.21	0.04	–6,376	0.21	0.04	–6,345	0.21	0.04	–6,209
Seeds per spike	2016	0.35	0.04	15,460	0.38	0.07	14,934	**0.48**	**0.05**	**12,383**	0.44	3.21	12,370	0.44	3.19	12,402	0.44	3.23	12,395
Seeds per spike	2017	0.24	0.04	14,651	0.22	0.06	14,216	**0.29**	**0.04**	**14,111**	0.25	1.24	14,215	0.25	1.22	14,201	0.26	1.27	14,215
**Shattering**	2016	0.24	0.04	1,097	0.22	0.06	955	**0.38**	**0.05**	**728**	0.36	0.05	752	0.36	0.05	765	0.36	0.05	762
**Shattering**	2017	0.27	0.04	245	0.14	0.05	177	**0.29**	**0.04**	**92**	0.28	0.04	134	0.28	0.04	123	0.28	0.04	124
Spike length (cm)	2016	0.24	0.04	9,833	0.26	0.07	9,510	**0.65**	**0.05**	**7,628**	0.60	0.27	7,621	0.60	0.27	7,645	0.60	0.27	7,642
Spike length (cm)	2017	0.16	0.03	9,992	0.14	0.04	9,667	**0.39**	**0.05**	**9,503**	0.35	0.26	9,534	0.34	0.24	9,484	0.33	0.24	9,498
**Spike yield (g)**	2016	0.28	0.04	–7,394	0.36	0.07	–7,266	**0.54**	**0.05**	**–6,110**	0.47	0.05	–5,994	0.48	0.05	–6,066	0.49	0.05	–6,071
**Spike yield (g)**	2017	0.18	0.03	–8,764	0.20	0.06	–8,585	**0.25**	**0.04**	**–8,553**	0.21	0.04	–8,374	0.20	0.04	–8,429	0.22	0.04	–8,405
Spikelet density	2016	0.33	0.05	–8,594	0.26	0.07	–8,381	**0.65**	**0.07**	**–7,134**	0.59	0.05	–7,100	0.59	0.05	–7,133	0.59	0.05	–7,137
Spikelet density	2017	0.26	0.04	–6,380	0.20	0.06	–6,239	**0.46**	**0.05**	**–6,303**	0.37	0.04	–6,195	0.38	0.04	–6,243	0.38	0.04	–6,261
Spikelets per inflorescence	2016	0.26	0.04	7,508	0.37	0.07	7,252	**0.45**	**0.05**	**5,955**	0.43	0.15	5,960	0.43	0.15	5,975	0.43	0.15	5,973
Spikelets per inflorescence	2017	**0.21**	**0.04**	**8,409**	0.20	0.06	8,192	0.21	0.05	8,132	0.18	0.10	8,206	0.19	0.11	8,226	0.19	0.11	8,252
Stem angle	2016	0.42	0.05	21,927	0.45	0.08	21,396	**0.60**	**0.06**	**17,561**	0.48	1.62	17,523	0.47	1.63	17,555	0.47	1.64	17,567
Stem diameter (mm)	2016	**0.44**	**1.57**	**–789**	0.29	0.13	–803	0.31	0.05	–720	0.33	0.05	–723	0.32	0.05	–714	0.32	0.05	–712
Stem strength bottom	2016	0.27	0.06	18,225	0.14	0.10	17,685	**0.39**	**0.14**	**14,634**	0.31	6,727.71	14,635	0.30	6,692.59	14,636	0.31	6,766.45	14,635
Stem strength middle	2016	0.25	0.04	16,707	0.08	0.07	16,192	**0.35**	**0.08**	**13,409**	0.31	2,049.1	13,410	0.31	2,030.71	13,409	0.31	2,056.5	13,409
Stem strength top	2016	NA	NA	NA	0.71	0.28	13,579	**0.89**	**0.07**	**11,224**	0.26	231.23	11,225	0.27	236.01	11,218	0.27	233.92	11,216
Minimum value		0.05	0.02	–13,300	0.01	0.02	–13,010	0.11	0.03	–13,094	0.09	0.03	–12,880	0.09	0.03	–12,940	0.10	0.03	–12,894
Maximum value		0.83	2.59	21,927	0.71	0.28	21,396	0.89	0.14	19,172	0.63	6,727.71	19,257	0.66	6,692.59	19,298	0.65	6,766.45	19,302
Number for the best model for trait^b^		9	42	12	0	0	1	33	2	21	2	1	7	2	0	3	0	1	2
Percent for best model		20%	91%	26%	0%	0%	2%	72%	4%	46%	4%	2%	15%	4%	0	7%	0	2%	4%

^a^Priority traits names are in bold, and the model with best fit for heritability is also in bold.

^b^ Number for the best model is number of times model had the highest heritability estimate or lowest SE or AIC for 46 traits.

### 3.2 Effect of GS models

We found little evidence that the choice of GS model significantly impacted GS predictive ability, with the simple GBLUP model having the highest predictive ability for 45 of 46 traits. Across the seven different models and a 20‐fold cross‐validation sampling procedure, predictive ability for all traits ranged between 0.78 to 0.98. Priority trait predictive abilities ranged from 0.89 for spike yield to 0.96 for free threshing, shattering, and seed mass. For all individual traits, the models were quite similar, with the maximum difference between the highest and lowest performing models for any one trait being 0.036. The average difference between the highest and lowest performing models was 0.016 (Supplemental Table S2). For 45 of 46 traits, the correlations between GEBVs for all GS models were significant, with all correlations above *r *= .97 (Supplemental Figure S1). Given the near equal performance, in terms of practical application, all subsequent GS results are from the GBLUP model fitted by the *rrBLUP* package.

Genomic predictions were affected by the choice of phenotypic adjustment. In light of the high correlations between Model 3 BLUPs and GEBVs, we investigated GS predictive ability for BLUPs from Models 1 and 2. These models did not use the GRM to calculate adjusted phenotypes, removing any potential bias of using the same markers to adjust the phenotype and predict the GS response. Across all traits, a similar pattern emerged that GS results based on Model 3 had higher correlations than those based on Model 2, and both were higher than Model 1, (Table 2, Supplemental Figure S2), where predictive ability is the correlation between the GS GEBVs and the model BLUPs for each particular model (i.e., Model 3 BLUPs were correlated to GEBVs developed from Model 3 BLUPs). Model 1 had much lower predictive abilities (−0.05 to 0.33, Table 2) than Model 3, which ranged from 0.80 to 0.98. This trend also extended to the observed phenotypes, where Model 3 always had higher correlations to the observed phenotypes than Models 1 or 2.

**TABLE 2:** Genomic selection (GS) predictive abilities for three different phenotypic evaluations. A 20‐fold cross‐validation was used for all GS model evaluations. Correlation between GS genomic estimated breeding values (GEBV) and model best linear unbiased predictors (BLUP) as well as correlation to observed phenotypes are reported for each model. Priority traits for selection are shown in bold

		Predictive ability for model BLUP and GS GEBV			Correlation of observed, uncorrected phenotype and GS GEBV		
Trait	Year	Model 1^a^	Model 2	Model 3	Model 1	Model 2	Model 3
Flag leaf height	2016	0.12	0.83	0.87	.25	.40	.55
Flag leaf length	2016	0.15	0.80	0.94	.26	.35	.52
Flag leaf width	2016	0.24	0.82	0.91	.37	.45	.62
Florets per spikelet	2016	0.09	0.81	0.91	.17	.32	.49
Florets per Spikelet	2017	0.08	0.88	0.98	.12	.18	.32
**Free threshing** ^b^	**2016**	**0.20**	**0.86**	**0.94**	**.50**	**.59**	**.67**
**Free threshing**	**2017**	**0.18**	**0.86**	**0.96**	**.39**	**.49**	**.61**
Maturity	2016	0.32	0.85	0.91	.42	.52	.68
Number of florets per spike	2016	0.17	0.79	0.91	.27	.37	.54
Number of florets per spike	2017	0.01	0.87	0.97	.10	.23	.34
Percent seed set	2016	0.08	0.83	0.93	.24	.37	.50
Percent seed set	2017	0.06	0.85	0.96	.17	.29	.43
Percent spike emergence	2017	0.18	0.81	0.93	.31	.44	.58
Plant height	2016	0.19	0.81	0.80	.27	.39	.56
Plant height	2017	0.17	0.83	0.91	.26	.28	.53
Seed area	2016	0.19	0.87	0.92	.42	.58	.67
Seed area	2017	0.09	0.87	0.96	.28	.41	.50
Seed density	2016	0.15	0.85	0.91	.28	.37	.52
Seed density	2017	0.12	0.86	0.95	.23	.37	.46
Seed image circularity	2016	0.28	0.87	0.91	.48	.58	.69
Seed image circularity	2017	0.22	0.84	0.93	.32	.36	.53
Seed length	2016	0.27	0.86	0.91	.46	.65	.72
Seed length	2017	0.20	0.85	0.94	.40	.56	.66
**Seed mass**	**2016**	**0.17**	**0.87**	**0.92**	**.36**	**.48**	**.60**
**Seed mass**	**2017**	**0.15**	**0.88**	**0.96**	**.35**	**.47**	**.57**
Seed perimeter	2016	0.27	0.87	0.91	.46	.64	.72
Seed perimeter	2017	0.17	0.86	0.95	.37	.54	.63
Seed width	2016	0.21	0.86	0.92	.42	.51	.64
Seed width	2017	0.09	0.87	0.97	.21	.29	.39
Seeds per spike	2016	0.13	0.83	0.92	.33	.47	.59
Seeds per spike	2017	0.08	0.84	0.95	.23	.35	.50
**Shattering**	**2016**	**0.15**	**0.85**	**0.94**	**.34**	**.41**	**.55**
**Shattering**	**2017**	**0.10**	**0.87**	**0.96**	**.30**	**.40**	**.54**
Spike length	2016	0.33	0.81	0.86	.40	.44	.62
Spike length	2017	0.20	0.85	0.94	.26	.31	.48
**Spike yield**	**2016**	**0.22**	**0.81**	**0.90**	**.34**	**.45**	**.58**
**Spike yield**	**2017**	**0.08**	**0.80**	**0.95**	**.18**	**.29**	**.45**
Spikelet density	2016	0.26	0.81	0.86	.37	.45	.62
Spikelet density	2017	0.14	0.83	0.92	.25	.35	.52
Spikelets per inflorescence	2016	0.16	0.77	0.90	.28	.43	.57
Spikelets per inflorescence	2017	–0.05	0.81	0.95	.06	.32	.45
Stem angle	2016	0.23	0.82	0.94	.38	.49	.64
Stem diameter	2016	0.11	0.85	0.94	.28	.42	.54
Stem strength bottom	2016	0.08	0.84	0.88	.20	.31	.47
Stem strength middle	2016	0.12	0.86	0.90	.25	.34	.49
Stem strength top	2016	0.14	0.86	0.91	.22	.30	.46
Minimum		–0.05	0.77	0.80	.06	.18	.32
Mean		0.16	0.84	0.92	.30	.41	.55
Maximum		0.33	0.88	0.98	.50	.65	.72

^a^ Model 1 is spatial correction with row and column first‐order autoregression (AR1) and random genet term. Model 2 is spatial correction with row and column AR1 and the additive relationship matrix (**A**). Model 3 is the same as Model 2, except **A** is replaced with the genomic realized relationship matrix**, GRM**.

^b^ Priority traits are in bold.

### 3.3 Factors effecting GS accuracy

#### 3.3.1 Marker number and training population size

Changing size of the training population or the number of markers had large effects on predictive ability. Across all traits, similar trends were evident that as training population size or marker number increased, predictive ability increased. As the size of the training population increased from 250 to 2,000 individuals, predictive ability for each trait increased by 0.15 on average (range: 0.08–0.26), with the sharpest increases observed with fewer individuals in the training population (Supplemental Figure S3). The largest gain in predictive ability was 0.26 for plant height, whereas free threshing showed the minimum gain in predictive ability of only 0.08 as the training size increased from 250 to 1,500 genets.

**FIGURE 2:**
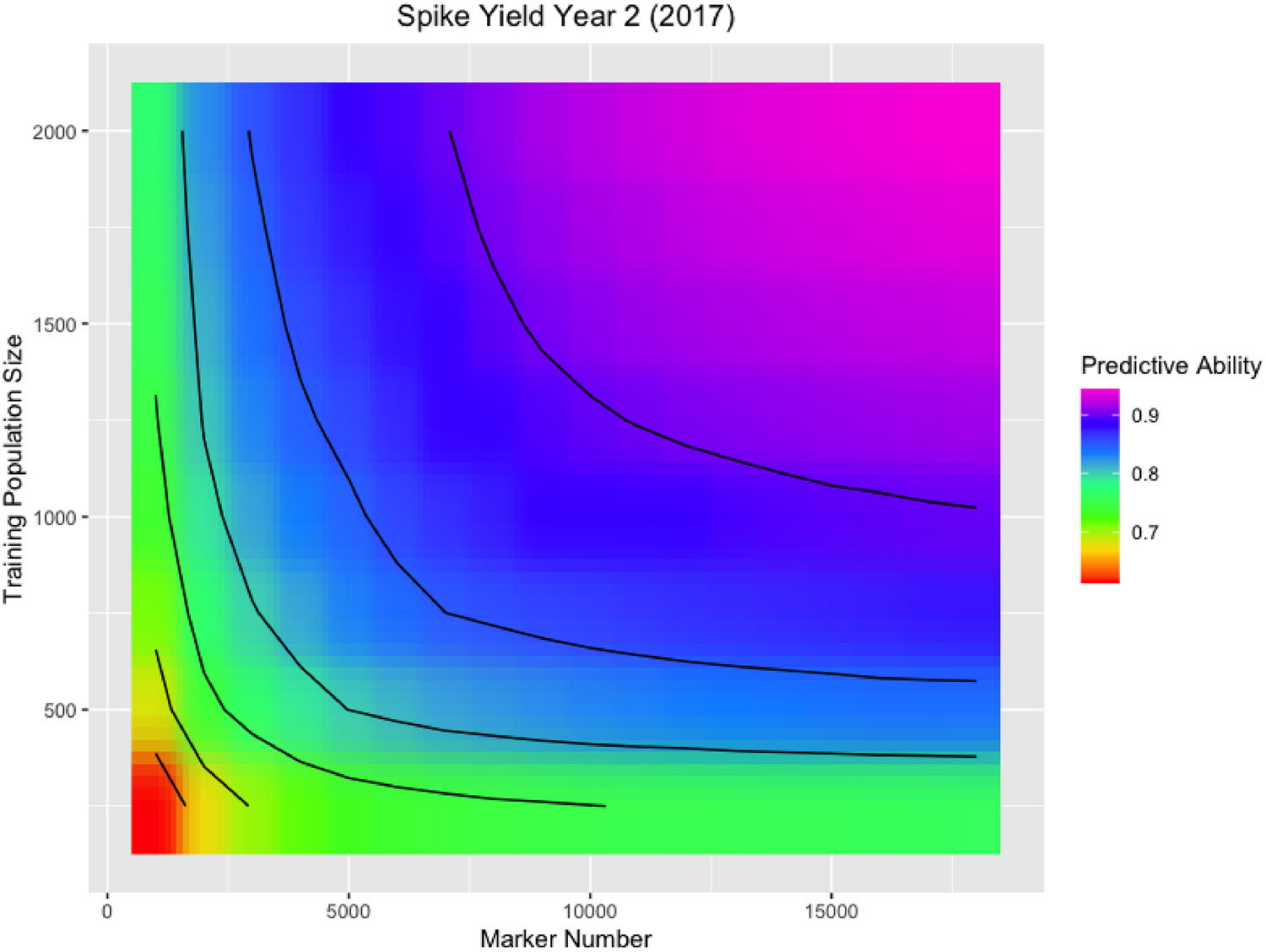
Contour plot of genomic selection (GS) predictive ability for differing levels of markers (*x* axis) and number of individuals (*y* axis) in the training population. Predictive ability is the average value of 100 iterations at each respective level, with contour lines representing differences of 0.05 in predictive ability

Increasing marker number also increased predictive ability, with a mean increase in predictive ability across all traits of 0.27 from increasing the marker number from 250 to 17,500. The largest difference for any trait (spike length) resulted in an increase of 0.38, with a minimum increase of 0.19 for free threshing. Predictive ability increased rapidly for the first 250 to 1,500 markers and more slowly with the addition of more markers (Supplemental Figure S3). Along with increasing predictive ability, the variance among simulations decreased as marker and training population size increased. Although there were clearly diminishing returns as marker number and training population size increased, we did not reach the asymptote in predictive ability at the levels tested. Taken together, both the number of markers and the size of the training population affected predictive ability (Figure 2) with a linear model including quadratic factors and the interaction for marker number and training population accounting for 94% of the observed variation in predictive ability.

#### 3.3.2 Training population composition

The choice of partitioning the dataset in different *k*‐fold cross‐validations had a minimal impact on predictive ability. Fitting 100 iterations of *k* (5, 10, 15, or 20) fold cross‐validation where 80, 90, 93, or 95% of the data was used to predict the remaining set, we found very little difference in the *k*‐fold validation (e.g., Supplemental Figure S4). There was a trend that a five‐fold cross‐validation had lower predictive ability, with increasing predictive ability at higher cross‐validations, but across all 46 traits, the average difference in a 5‐ and 20‐fold cross‐validation was 0.01, with a maximum difference of any trait for plant height in 2016 of 0.02 (five‐fold cross‐validation: *r *= .79; 20‐fold cross‐validation: *r* = .81). Our choice of a 20‐fold validation appears not to have significantly inflated predictive ability.

**FIGURE 3:**
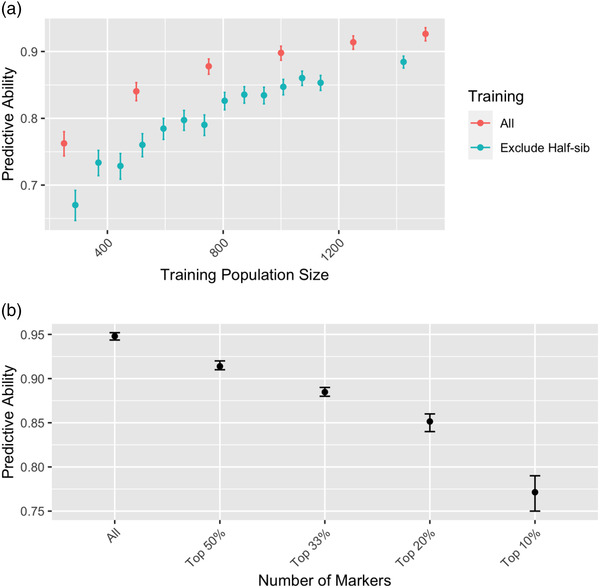
Effect of training population composition (Panel A) and marker number (Panel B) on genomic selection (GS) predictive ability for spike yield 2017. In Panel A, the training population was made from either randomly partitioning all genets, or half‐sib training populations, where, for each half‐sib family predicted, the common genotype was excluded from the training population. In Panel B, GS predictive ability was evaluated with 18,357 (all) markers compared with varying levels of markers with the fewest missing data. The 95% confidence intervals for the correlation between best linear unbiased predictors and genomic estimated breeding values for each level are shown

Overall, there was a significant trend that predictions made by excluding half‐sib members were lower than those with randomly drawn sets (Figure 3). Across all traits, the difference in predictive ability was greatest for the smallest training population, up to a 0.08 difference on average. As training population increased from 1,000 to 1,500 individuals, the average difference between randomly drawn and family‐specific training populations dropped from 0.05 to 0.02. This result suggests that large training populations can make accurate predictions regardless of known family composition.

Developing GS models based on the amount of missing data per marker showed a significant trend that more markers, even with higher levels of missing data, provided higher GS predictive ability (Figure 3). Higher predictive abilities were always observed when more markers were added to the model, suggesting that within this population, having larger marker numbers is beneficial, even with increasing amounts of missing data.

### 3.4 Potential for genetic gain

**FIGURE 4:**
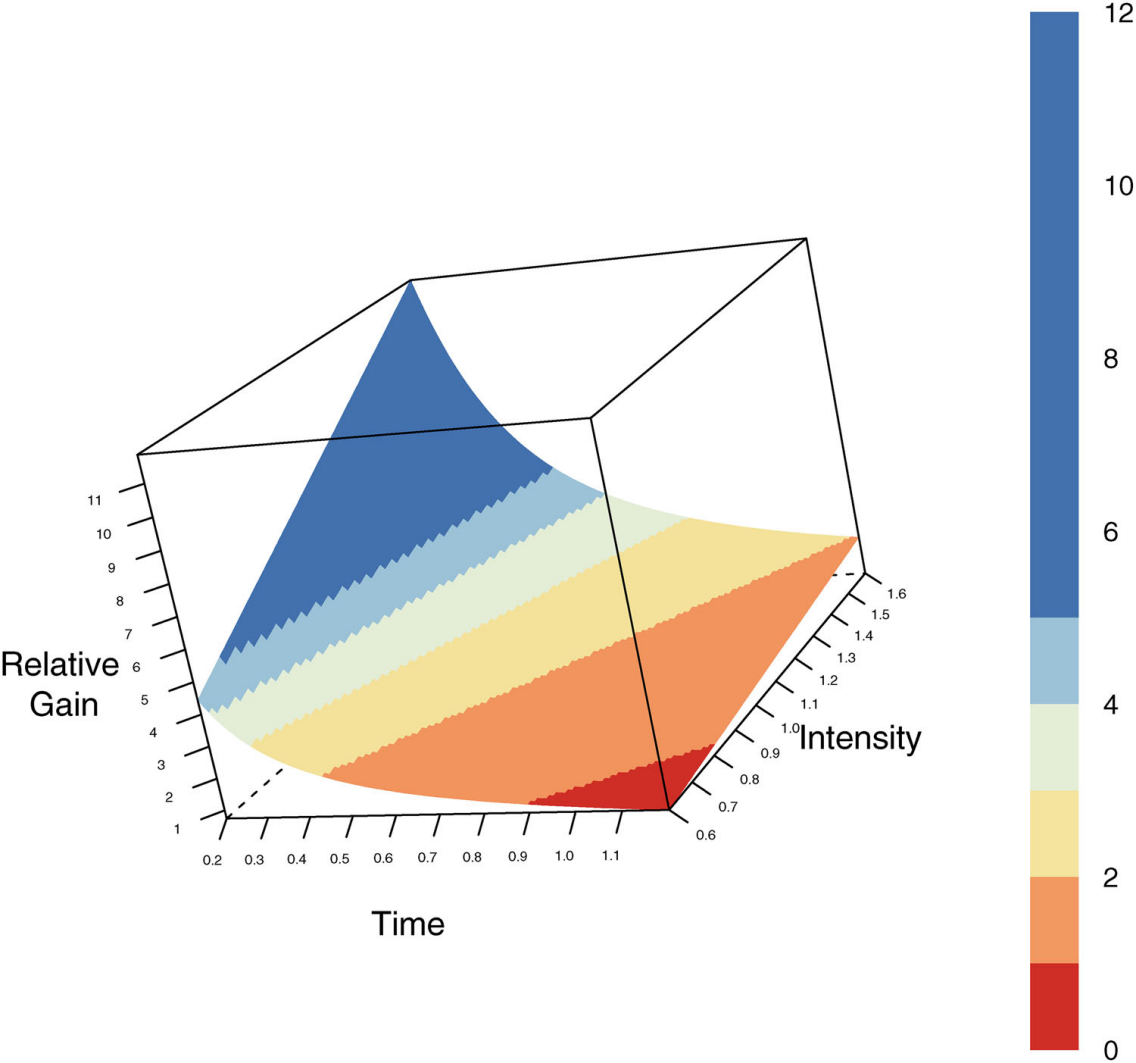
Relative efficiency of genetic gain for genomic selection (GS) compared with direct phenotypic selection (PS) for spike yield. Relative gain is on the *z* axis, with the variables of program time (*x* axis) and selection intensity (*y* axis). Program time is compared with PS, where 1 indicates that the same time was needed to complete both GS and PS; values lower than 1 indicate that GS was completed faster than PS (i.e., 0.5 means that GS takes half the time of PS). Selection intensity is the ratio of the intensity of selection, *i*, for GS and PS. An intensity of 1 indicates the same selection intensity under both GS and PS, where values lower than 1 are lower selection intensities for GS than PS

We calculated the relative genetic gain when selecting a correlated trait (e.g., genomic prediction) relative to direct selection of the phenotype for a range of population sizes, predictive abilities, heritability, and relative cycle time (Figure 4) and found strong support for an overall increased breeding program rate of genetic gain with GS. Largely driven by reduced cycle time at the extreme values, if GS can be completed five times faster than PS, the relative gains from GS could exceed PS genetic gains by 10‐fold. With the expected program resources, in which the selection intensity for a GS program is lower than PS (0.87) but the GS interval time is one half of PS, we estimate that GS can provide up to a sevenfold increase per unit time compared with PS (Table 3). For priority traits, the estimated gains were 2.6‐, 6.6‐, 3.8‐, and 5.3‐fold in spike yield, seed mass, free threshing, and shattering, respectively. These estimated rates of gain suggest that GS is an attractive breeding method to quickly improve IWG.

**TABLE 3:** Relative efficiency of selection via indirect (genomic selection, GS) versus direct phenotypic selection (PS) for intermediate wheatgrass traits, with selection intensities (*i*) of 1% for PS (*i* = 2.665) and 2.5% selection intensity for GS (*i* = 2.338). Priority traits are shown in bold

Trait	Narrow‐sense heritability	*r* ^a^ *_prediction_*	CR/R^b^
Florets per spikelet	0.21	.41	3.4
**Free threshing** ^c^	**0.39**	**.83**	**3.8**
Number of florets per spike	0.30	.54	3.2
Percent seed set	0.25	.56	4.0
Plant height	0.24	.46	3.3
Seed area	0.37	.80	3.8
Seed density	0.13	.52	7.1
Seed image circularity	0.30	.76	4.5
Seed length	0.45	.84	3.3
**Seed mass**	**0.19**	**.71**	**6.6**
Seed perimeter	0.43	.84	3.4
Seed width	0.26	.70	4.7
Seeds per spike	0.38	.66	3.1
**Shattering**	**0.22**	**.66**	**5.3**
Spike length	0.26	.64	4.3
**Spike yield**	**0.36**	**.53**	**2.6**
Spikelet density	0.26	.61	4.1
Spikelets per inflorescence	0.37	.62	2.9
Max.	0.39	.83	7.1
Min.	0.21	.41	2.6

^a^ Correlation between GS genomic estimated breeding value for 2016, formed from 20‐fold cross‐validation, and best linear unbiased prediction for 2017.

^b^ Response to selection using GS as a correlated trait (*CR*) compared with direct phenotypic selection (*R*).

^c^ Priority traits are in bold.

## 4 DISCUSSION

### 4.1 Application of spatial correction

The IWG breeding program has historically used recurrent selection, where the best potential parents are identified from field evaluations of thousands of unreplicated genets. We evaluated several potential models to best assess genotypic IWG trait performance along with spatial heterogeneity. As the breeding program moves toward more emphasis on molecular breeding (GS), accurate phenotypes are needed to develop training models (Cobb, DeClerck, Greenberg, Clark, & McCouch, 2013; Meuwissen, Hayes, & Goddard, 2001), and the genetic gains from breeding will be proportional to the accuracy of field assessment (Cobb et al., 2013, 2019).

Testing two different types of models (AR1 and moving means covariate) on the TLI breeding data showed that AR1 models consistently outperformed other models. Additionally, the AR1 row and column correction can be used with minimal manual tuning, which is very beneficial for pipelined analysis. Although other studies, including Lado et al. (2013) and Gezan, White, & Huber (2010), have shown benefits of moving grid adjustment, the single genets at close spacing used in the IWG program may affect the results more than yield plots, which are often multiple meters in each direction. With IWG, the nearest plants, equivalent to plots, were only 0.6 m away, and at a 1.5‐m moving grid, only a few plants close together would be used to assess the local environment. At such small spatial levels, random error may obscure the true spatial trend. Though our results advocate for the AR1 correction, similar to Gezan et al. (2010); Leiser, Rattunde, Piepho, & Parzies (2012); and Gilmour et al. (1997), breeding programs should carefully assess the environment and experimental procedures to obtain the best possible predictions of plant phenotype.

### 4.2 Effect of GS models

The initial GS results across all traits produced extremely high cross‐validation predictive abilities and were consistent across different prediction models. Unlike Zhang et al. (2016), we did not see an increase in predictive ability with the Gaussian kernel models. This was potentially attributed to nonadditive effects and may reflect that their training population was made of 66 half‐sib families. In the present study, along with the Gaussian kernel, we did not see improved predictive ability with the GBLUP dominance model, which should also capture nonadditive effects. As IWG is heterozygous and, in theory, dominance should be expected, it is likely that the formulation of our phenotypic adjustment (BLUPs) accounting for parent interaction accounted for most of the dominance effects. Although the predictive abilities with only additive methods are high, other research has shown that the inclusion of dominance effects into GS models can boost the accuracy of predictions (Toro & Varona, 2010; Wellmann & Bennewitz, 2012) and also maintain this accuracy across generations (Wellmann and Bennewitz, 2012). Recent research by Bajgain, Zhang, & Anderson (2020) has shown increases in GS predictive ability in IWG with dominance models. As the breeding program progresses, estimating dominance may become more vital. Since the models were also nearly identical in GS predictions for all traits, we chose the GBLUP model to use for further evaluation, as it is easy to implement (for the analyst) and fast (computing efficiency).

### 4.3 Factors affecting GS accuracy

The GS predictions were unexpectedly high, so we investigated several potential explanations. There is a wide range of genetic variation, which should result in high GS predictive ability, as GS model predictions decreases with decreasing genetic variance (Jannink, 2010). These analyses, therefore, were designed to identify any potential model bias that would inappropriately inflate GS predictions. First, we examined training population size. As the training population size increased, GS predictive ability increased. Yet even at low (250–500 individuals), the observed predictions were higher than those reported by Zhang et al. (2016), although our methods differed significantly in adjusting for field and genet effects. In Zhang et al. (2016), year effects were removed from observations; thus, our correlation to observed phenotypes (Table 2) is the closest comparison to their methods and are approximately similar in value. The size of our training population was routinely over 1,700 individuals (20‐fold cross‐validation), whereas the entire population size used by Zhang et al. (2016) was only 1,126 genets (only 65% or less of the size used in this study). Although large GS studies have been reported, most of the GS literature in plants is based on much smaller training populations. Rutkoski, Crain, Poland, and Sorrells (2017) reviewed over 40 GS experiments in published literature with population sizes ranging from 65 to 2,437 with a median size of 446 individuals, substantially fewer individuals than used in this study, with higher predictions in this study resulting from the large training population.

A second potential reason for the observed high GS predictions was the large number of markers used (18,357) compared with many studies in small grains (Rutkoski et al., 2017). As marker density increases across the genome, it is more likely that each quantitative trait locus will be linked with markers, increasing the predictive ability of GS models (Heffner, Sorrells, & Jannink, 2009; Meuwissen et al., 2001; Meuwissen, 2009). Across all traits, the number of markers followed a similarly increasing pattern, with large increases in predictions when up to 1,500–2,500 markers were used, then smaller increases up to the maximum marker number. However, even at low marker numbers (250–500), prediction values for spike yield and plant height were higher than those reported by Zhang et al. (2016) for 3,883 markers. Additionally, when markers were subdivided by missing data, there appeared to be no benefit to excluding markers with more missing data. This indicates that next‐generation sequencing techniques like GBS that produce lots of missing data can be as effective in terms of GS as more expensive array‐based data (Heslot, Rutoski, Poland, Jannink, & Sorrells, 2013; Elbasyoni et al., 2018).

Thirdly, we used the known parents of the TLI‐Cycle 6 population to predict genet performance. Predicting half‐sib families allowed predictions to be made without the potential bias of closely related individuals in both the training population and the prediction population. Although there was a significant trend that random training populations were upwardly biased (Figure 3), this effect was small at larger (>1,000 genets) training populations. This result suggests that the IWG breeding programs can focus directly on increasing the size of the training population, rather than selectively ensuring representation by specific crosses or families, as suggested by Zhang et al. (2016).

Finally, we also examined how the use of statistical models to account for field effects would impact GS. A model that leveraged genomic relationships and incorporated spatial correction (Model 3) was found to be the most robust and was used to calculate the BLUPs that were used to train and validate the GS model. The GRM was formed by the same markers that were used in the GS model, potentially confounding the results. To test this, we also ran GS models on BLUPs from Models 1 and 2. Model 1 did not use the pedigree or molecular markers, avoiding confounding across the phenotypic and GS model. Model 2 only accounted for the average relationship with the additive relationship matrix, where Model 3 should also account for random Mendelian sampling, which could vary substantially from the predicted values (Hill & Weir, 2011), thus resulting in a better fit. Our results confirmed this, with Model 3 resulting in higher GS predictions and correlations to both BLUPs and observed data (Table 2, Supplemental Figure S2). The use of the same molecular markers to calculate both the BLUPs and the GEBVs could be confounded, yet the data indicated that obtaining higher precision (heritability) (Isik et al., 2017) in the phenotypic adjustment will ultimately result in more accurate GS. As the IWG program progresses, this is an area that warrants further research to obtain the best possible results from applying GS in the IWG program, but, currently, it appears any potential confounding effects in Model 3 are offset by higher prediction accuracies, which should translate to higher genetic gains.

### 4.4 Applying GS in the IWG breeding program

**FIGURE 5:**
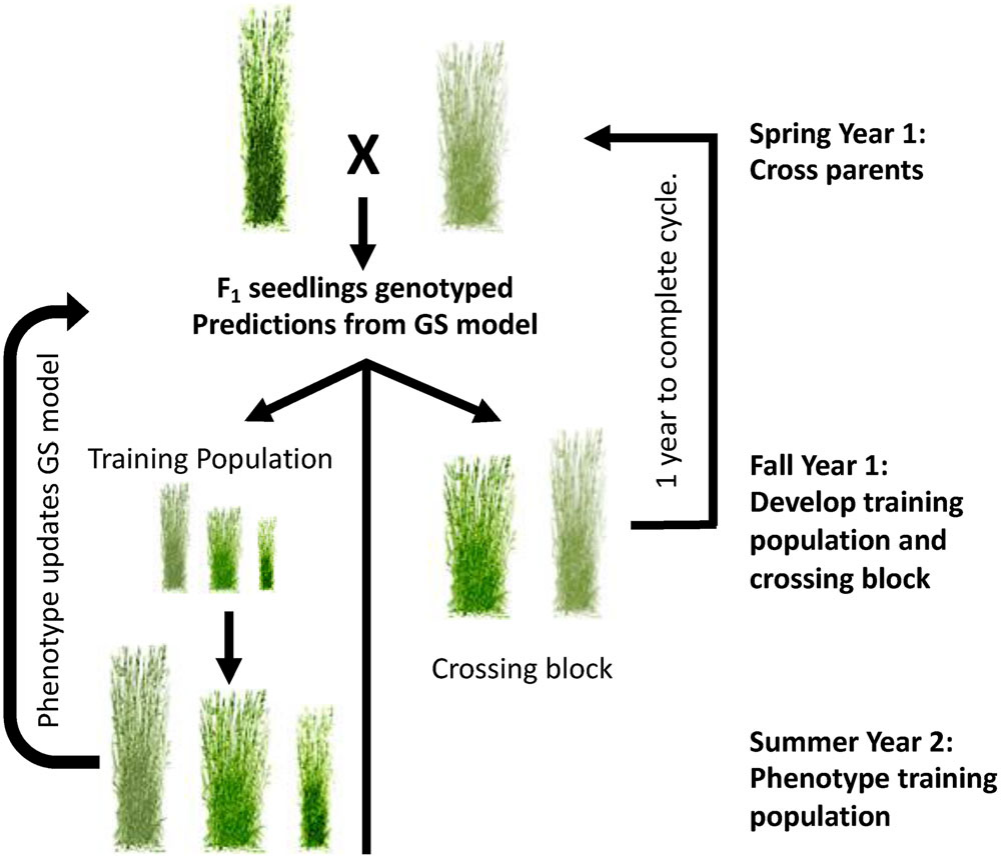
Proposed genomic selection (GS) breeding program that completes one breeding cycle per year

Over a range of traits and years, the results showed that GS could be used to accurately predict phenotypes targeted for selection. Although a breeding scheme using GS in IWG was proposed by Zhang et al. (2016), it still required 2 yr to complete a full cycle of selection. The benefits of this method were the cost savings associated with reduced field evaluations and increased selection intensity. We propose the following strategy, which allows a cycle of selection to be completed every year (Figure 5). By reducing the cycle time from 2 yr to 1 yr, expected genetic gains could be more than doubled, with the potential to further increase through higher selection intensity and measurement accuracy.

The proposed breeding program begins with starting (∼4,500) F_1_ seed from parent crosses in the greenhouse in late summer. At seedling stage, tissue sampling and genotype profiling are conducted on ∼4,000 individuals, with visual observation used to discard poor‐performing seedlings. The GS model is used to determine the best 100 genets, which are maintained in the greenhouse as the crossing block for the next cycle. Another 1,000 to 1,200 plants are transplanted in the field in late fall for phenotypic evaluation of these new genets, representing the current material in the breeding program. After field evaluations the following summer, the GS model can be updated for the subsequent cycle of plants that were generated from the 100 parent plants. In this fashion, a complete cycle is completed each year.

With this rapid cycle breeding, the training model for GS is one cycle behind the breeding population, but this can be continually updated on a yearly basis with new phenotype observations coming from the field. This breeding strategy also allows for multiple years of the observation of the training population while simultaneously recombining germplasm each year. We envision that up to 3 yr of phenotypic observations could be completed, informing the GS model, allowing three cycles of crossings to occur in the same timeframe as one cycle of 3 yr of phenotypic observation to investigate traits like sustained yield. In this scenario, the program would benefit from prolonged phenotypic observation, yet still make more progress than would be possible even under phenotypic selection with 1 yr of observation with no information about trait performance in the second and subsequent years.

### 4.5 Potential gains in the IWG breeding program from GS

One of the largest benefits of GS is decreasing the breeding cycle time (Heffner et al., 2009). Within the perennial IWG, it should be possible to decrease the cycle time by half at least compared with PS. The reduction in the breeding cycle could theoretically double the rate of genetic gains. Although the TLI breeding program is currently based on 2 yr per PS, early work often evaluated plants for two or more years by extending the PS cycle time and increasing the benefits of GS (Figure 4). Especially for evaluating long‐term phenotypes, such as stand persistence over years, GS could radically improve genetic gains compared with PS.

In addition to reducing cycle time, GS also allows a reduction in selection intensity. Under PS, up to 10,000 genets would be evaluated and only the top 1% would be selected. This strong selection intensity can drive breeding gains if the selection is accurate. If these selected plants are in error, either from optimal field location or data errors, major reductions (up to 77% lower in extreme cases) in genetic gains can occur and genetic gains can be improved by decreasing the selection intensity (Mackay & Caligari, 1999). Along with potentially decreased genetic gains, higher selection intensities lead to increased rates of inbreeding and loss of genetic diversity (Silvela, Rodgers, Barrera, & Alexander, 1989). As proposed within IWG, GS would allow for a selection intensity of 2.5%, decreasing the chance of inadvertently picking inferior genotypes and preventing excessive loss of genetic diversity caused by more intensive selection.

Combining the reduction in cycle time and selection intensity should allow GS to provide superior gains in the breeding program. Although we estimate up to a 2.6‐fold increase in spike yield, and even higher increases for other traits (Table 3), these estimates are based on one cycle and one data location. Consequently, these estimates should represent an upper bound of what could possibly be achieved. However, even if only half of the expected gains are achieved, GS could still outperform PS in terms of increasing genetic gain.

## 5 CONCLUSIONS

Breeding programs require large investment of resources to improve crops. The ability to increase the rate of genetic gain through genomic tools is vital to rapidly developing perennial crops like IWG. This research has evaluated how a breeding program could transition from PS to GS and the potential increase in genetic gain that could be achieved. Emphasis was placed on finding the best statistical models to obtain accurate phenotype (BLUP) estimates through spatial and genetic correction, with the row–column (AR1 × AR1) model with the GRM providing the best fit to the observed data. Genomic selection was evaluated, showing that GS models could accurately predict phenotypes, with predictive ability depending on the size of the training population and the number of markers used. Finally, we estimated that significant genetic gains, more than doubling PS gains, could be achieved from using GS in the IWG breeding program.

## AUTHOR CONTRIBUTIONS

JC, LD, and JP provided the conceptualization and methodology. LD conducted the investigations and provided plant resources. JC and AH conducted the formal analysis. JC prepared the original draft, with all authors reviewing, editing, and approving the final manuscript.

## CONFLICT OF INTEREST

The authors declare that they have no conflict of interest. The funding sponsors had no role in experimental design, data collection, analysis, interpretation of the results, or the decision to publish results.

## Supporting information


**Supplemental Table S1**. Summary statistics including minimum, maximum, average, and SD for traits measured during the 2016 and 2017 growing seasons for intermediate wheatgrass (Salina, KS).
**Supplemental Table S2**. Predictive ability as the correlation of model prediction and adjusted phenotypic value for several GS models for intermediate wheatgrass traits. Priority traits are in bold.
**Supplemental Figure S1**. Correlation matrix between observed and adjusted phenotype values and seven different GS models for spike yield in 2016.
**Supplemental Figure S2**. Correlation matrix between observed phenotypes, BLUPs (phenotypic adjustment), and GEBVs from GS models for spike yield in 2017.
**Supplemental Figure S3**. Genomic selection predictive ability for different marker numbers (Panel A) and the effect of training population size (Panel B) for spike yield in 2017.
**Supplemental Figure S4**. Boxplot for GS predictive ability using *k*‐fold cross‐validations of 5, 10, 15, and 20, for 100 random iterations for spike yield in 2017.
