# Supplementary material for: Development of whole‐genome prediction models to increase the rate of genetic gain in intermediate wheatgrass (Thinopyrum intermedium) breeding
Source: Plant Genome. 2021 Apr 26;14(2):e20089. doi: 10.1002/tpg2.20089 (PMC12807048; doi:10.1002/tpg2.20089)
Supplement: Supplementary file 1 [file TPG2-14-e20089-s001.docx]

Supplemental Material

**Supplemental Table S1**. Summary statistics including minimum, maximum, average, and standard deviation for traits measured during the 2016 and 2017 growing seasons for intermediate wheatgrass, Salina, KS. Priority traits are in bold.

| **Trait** | **Year** | **n** | **min** | **max** | **mean** | **stdev** |
| --- | --- | --- | --- | --- | --- | --- |
| Flag Leaf Height (cm) | 2016 | 1741 | 10 | 97 | 64.32 | 11.2 |
| Flag Leaf Length (cm) | 2016 | 2595 | 7 | 53 | 27.65 | 5.18 |
| Flag Leaf Width (mm) | 2016 | 2594 | 4 | 34 | 15.68 | 2.98 |
| Florets per Spikelet | 2016 | 2275 | 1 | 12 | 6.98 | 1.49 |
| Florets per Spikelet | 2017 | 2354 | 3 | 12 | 5.89 | 1.39 |
| **Free Threshing %** | **2016** | **2242** | **0** | **100** | **41.23** | **23.3** |
| **Free Threshing %** | **2017** | **2354** | **0** | **100** | **26.22** | **20.62** |
| Maturity | 2016 | 2930 | 51 | 69 | 59.76 | 4.13 |
| Number of Florets per Spike | 2016 | 2272 | 24 | 324 | 166.91 | 45.16 |
| Number of Florets per Spike | 2017 | 2355 | 0 | 320 | 140.86 | 44.64 |
| Percent Seed Set | 2016 | 2227 | 0 | 1.34 | 0.22 | 0.12 |
| Percent Seed Set | 2017 | 2330 | 0 | 0.78 | 0.25 | 0.13 |
| Percent Spike Emergence | 2017 | 2353 | 0 | 2.56 | 0.75 | 0.31 |
| Plant Height (cm) | 2016 | 1738 | 30 | 180 | 113.77 | 15.59 |
| Plant Height (cm) | 2017 | 2367 | 55 | 175 | 128.12 | 14.15 |
| Seed Area (mm^2^) | 2016 | 2240 | 3.87 | 12.38 | 8.31 | 1.21 |
| Seed Area (mm^2^) | 2017 | 2355 | 3.87 | 14.11 | 7.23 | 1.47 |
| Seed Density | 2016 | 2240 | 0 | 1.49 | 0.8 | 0.16 |
| Seed Density | 2017 | 2354 | 0.43 | 1.85 | 0.91 | 0.16 |
| Seed Image Circularity | 2016 | 2240 | 0.37 | 0.68 | 0.49 | 0.04 |
| Seed Image Circularity | 2017 | 2355 | 0.34 | 0.63 | 0.47 | 0.04 |
| Seed Length (mm) | 2016 | 2240 | 4.31 | 8.24 | 6.34 | 0.59 |
| Seed Length (mm) | 2017 | 2355 | 4.17 | 8.92 | 6.15 | 0.61 |
| **Seed Mass (mg)** | **2016** | **2239** | **0.83** | **13.33** | **6.74** | **1.88** |
| **Seed Mass (mg)** | **2017** | **2354** | **2.58** | **13.73** | **6.58** | **1.62** |
| Seed Perimeter (mm) | 2016 | 2240 | 10.24 | 18.59 | 14.54 | 1.27 |
| Seed Perimeter (mm) | 2017 | 2355 | 9.61 | 20.12 | 13.88 | 1.37 |
| Seed Width (mm) | 2016 | 2240 | 1.13 | 2.16 | 1.69 | 0.14 |
| Seed Width (mm) | 2017 | 2355 | 1 | 2.14 | 1.54 | 0.18 |
| Seeds per Spike | 2016 | 2278 | 0 | 139.67 | 34.18 | 19.46 |
| Seeds per Spike | 2017 | 2353 | 0.26 | 101.22 | 33.38 | 14.72 |
| **Shattering** | **2016** | **2283** | **0** | **4** | **1.97** | **0.82** |
| **Shattering** | **2017** | **2363** | **0** | **5** | **2.44** | **0.67** |
| Spike Length (cm) | 2016 | 2280 | 13 | 60 | 39.06 | 5.42 |
| Spike Length (cm) | 2017 | 2353 | 12 | 56 | 30.45 | 5.57 |
| **Spike Yield (g)** | **2016** | **2282** | **0** | **0.81** | **0.23** | **0.13** |
| **Spike Yield (g)** | **2017** | **2368** | **0** | **0.73** | **0.22** | **0.11** |
| Spikelet Density | 2016 | 2277 | 0.21 | 1.25 | 0.62 | 0.1 |
| Spikelet Density | 2017 | 2348 | 0.33 | 2 | 0.8 | 0.17 |
| Spikelets per Inflorescence | 2016 | 2278 | 9 | 34 | 23.81 | 3.28 |
| Spikelets per Inflorescence | 2017 | 2363 | 12 | 43 | 23.8 | 3.8 |
| Stem Angle | 2016 | 3404 | 10 | 90 | 58.77 | 16.49 |
| Stem Diameter (mm) | 2016 | 2277 | 1 | 6 | 3.02 | 0.53 |
| Stem Strength Top | 2016 | 1275 | 44 | 1094 | 417.93 | 147.74 |
| Stem Strength Bottom | 2016 | 1274 | 252 | 5481 | 2158.22 | 795.58 |
| Stem Strength Middle | 2016 | 1275 | 123 | 3204 | 1109.8 | 436.99 |

**Supplemental Table S2.** Predictive ability as the correlation of model prediction and adjusted phenotypic value, for several genomic selection models for intermediate wheatgrass traits. Priority traits are in bold.

|  |  |  | Genomic Selection Models | | | | | | |  |
| --- | --- | --- | --- | --- | --- | --- | --- | --- | --- | --- |
| **Trait ID** | **Year** | **n^†^** | **GBLUP** | **GAUS** | **GBLUP Dominance** | **LASSO** | **BayesA** | **BayesB** | **BayesCπ** | **CI^‡^** |
| Flag Leaf Height | 2016 | 1387 | 0.87 | 0.86 | 0.87 | 0.85 | 0.86 | 0.86 | 0.86 | 0.013 |
| Flag Leaf Length | 2016 | 2117 | 0.94 | 0.92 | 0.93 | 0.93 | 0.93 | 0.93 | 0.93 | 0.005 |
| Flag Leaf Width | 2016 | 2117 | 0.91 | 0.90 | 0.91 | 0.90 | 0.90 | 0.90 | 0.90 | 0.007 |
| Florets per Spikelet | 2016 | 1834 | 0.91 | 0.89 | 0.9 | 0.90 | 0.90 | 0.90 | 0.90 | 0.008 |
| Florets per Spikelet | 2017 | 2281 | 0.98 | 0.97 | 0.97 | 0.97 | 0.97 | 0.97 | 0.97 | 0.002 |
| **Free Threshing** | **2016** | **1816** | **0.94** | **0.94** | **0.94** | **0.93** | **0.94** | **0.94** | **0.94** | **0.005** |
| **Free Threshing** | **2017** | **2280** | **0.96** | **0.95** | **0.95** | **0.95** | **0.96** | **0.95** | **0.95** | **0.003** |
| Maturity | 2016 | 2381 | 0.91 | 0.90 | 0.90 | 0.89 | 0.90 | 0.89 | 0.89 | 0.007 |
| Number of Florets per Spike | 2016 | 1832 | 0.91 | 0.89 | 0.90 | 0.89 | 0.90 | 0.89 | 0.90 | 0.008 |
| Number of Florets per Spike | 2017 | 2282 | 0.97 | 0.96 | 0.97 | 0.97 | 0.97 | 0.97 | 0.97 | 0.002 |
| Percent Seed Set | 2016 | 1803 | 0.93 | 0.92 | 0.92 | 0.92 | 0.92 | 0.92 | 0.92 | 0.007 |
| Percent Seed Set | 2017 | 2257 | 0.96 | 0.95 | 0.95 | 0.95 | 0.95 | 0.95 | 0.95 | 0.003 |
| Percent Spike Emergence | 2017 | 2279 | 0.93 | 0.91 | 0.92 | 0.92 | 0.92 | 0.92 | 0.92 | 0.006 |
| Plant Height | 2016 | 1384 | 0.80 | 0.81 | 0.81 | 0.78 | 0.78 | 0.78 | 0.78 | 0.019 |
| Plant Height | 2017 | 2293 | 0.91 | 0.90 | 0.91 | 0.89 | 0.90 | 0.90 | 0.90 | 0.007 |
| Seed Area | 2016 | 1814 | 0.92 | 0.91 | 0.92 | 0.91 | 0.91 | 0.91 | 0.91 | 0.007 |
| Seed Area | 2017 | 2281 | 0.96 | 0.95 | 0.96 | 0.96 | 0.96 | 0.96 | 0.96 | 0.003 |
| Seed Density | 2016 | 1814 | 0.91 | 0.89 | 0.90 | 0.90 | 0.90 | 0.90 | 0.90 | 0.008 |
| Seed Density | 2017 | 2280 | 0.95 | 0.94 | 0.95 | 0.95 | 0.95 | 0.95 | 0.95 | 0.004 |
| Seed Image Circularity | 2016 | 1814 | 0.91 | 0.89 | 0.90 | 0.90 | 0.90 | 0.9 | 0.90 | 0.009 |
| Seed Image Circularity | 2017 | 2281 | 0.93 | 0.91 | 0.92 | 0.92 | 0.92 | 0.92 | 0.92 | 0.006 |
| Seed Length | 2016 | 1814 | 0.91 | 0.90 | 0.90 | 0.90 | 0.90 | 0.90 | 0.90 | 0.008 |
| Seed Length | 2017 | 2281 | 0.94 | 0.93 | 0.94 | 0.94 | 0.94 | 0.94 | 0.94 | 0.005 |
| **Seed Mass** | **2016** | **1813** | **0.92** | **0.91** | **0.92** | **0.92** | **0.92** | **0.92** | **0.92** | **0.007** |
| **Seed Mass** | **2017** | **2280** | **0.96** | **0.95** | **0.95** | **0.95** | **0.95** | **0.95** | **0.95** | **0.004** |
| Seed Perimeter | 2016 | 1814 | 0.91 | 0.90 | 0.91 | 0.90 | 0.90 | 0.90 | 0.90 | 0.008 |
| Seed Perimeter | 2017 | 2281 | 0.95 | 0.94 | 0.94 | 0.94 | 0.94 | 0.94 | 0.94 | 0.004 |
| Seed Width | 2016 | 1814 | 0.92 | 0.91 | 0.91 | 0.91 | 0.91 | 0.91 | 0.91 | 0.008 |
| Seed Width | 2017 | 2281 | 0.97 | 0.95 | 0.96 | 0.96 | 0.96 | 0.96 | 0.96 | 0.003 |
| Seeds per Spike | 2016 | 1838 | 0.92 | 0.91 | 0.92 | 0.90 | 0.91 | 0.91 | 0.91 | 0.007 |
| Seeds per Spike | 2017 | 2279 | 0.95 | 0.94 | 0.94 | 0.94 | 0.94 | 0.94 | 0.94 | 0.004 |
| **Shattering** | **2016** | **1842** | **0.94** | **0.93** | **0.93** | **0.93** | **0.93** | **0.93** | **0.93** | **0.006** |
| **Shattering** | **2017** | **2289** | **0.96** | **0.95** | **0.95** | **0.96** | **0.96** | **0.95** | **0.95** | **0.003** |
| Spike Length | 2016 | 1839 | 0.86 | 0.85 | 0.86 | 0.85 | 0.85 | 0.85 | 0.85 | 0.012 |
| Spike Length | 2017 | 2279 | 0.94 | 0.92 | 0.93 | 0.92 | 0.93 | 0.93 | 0.93 | 0.005 |
| **Spike Yield** | **2016** | **1842** | **0.90** | **0.89** | **0.90** | **0.89** | **0.89** | **0.89** | **0.89** | **0.009** |
| **Spike Yield** | **2017** | **2294** | **0.95** | **0.93** | **0.94** | **0.94** | **0.94** | **0.94** | **0.94** | **0.004** |
| Spikelet Density | 2016 | 1837 | 0.86 | 0.85 | 0.86 | 0.85 | 0.85 | 0.85 | 0.85 | 0.012 |
| Spikelet Density | 2017 | 2274 | 0.92 | 0.90 | 0.91 | 0.91 | 0.91 | 0.91 | 0.91 | 0.006 |
| Spikelets per Inflorescence | 2016 | 1838 | 0.90 | 0.88 | 0.88 | 0.89 | 0.89 | 0.88 | 0.89 | 0.009 |
| Spikelets per Inflorescence | 2017 | 2289 | 0.95 | 0.93 | 0.94 | 0.94 | 0.94 | 0.94 | 0.94 | 0.004 |
| Stem Angle | 2016 | 2765 | 0.94 | 0.92 | 0.93 | 0.93 | 0.93 | 0.93 | 0.93 | 0.004 |
| Stem Diameter | 2016 | 1837 | 0.94 | 0.93 | 0.94 | 0.93 | 0.93 | 0.93 | 0.93 | 0.005 |
| Stem Strength Top | 2016 | 1026 | 0.91 | 0.90 | 0.90 | 0.89 | 0.90 | 0.90 | 0.90 | 0.011 |
| Stem Strength Bottom | 2016 | 1025 | 0.88 | 0.86 | 0.87 | 0.85 | 0.86 | 0.86 | 0.87 | 0.015 |
| Stem Strength Middle | 2016 | 1026 | 0.90 | 0.89 | 0.89 | 0.89 | 0.89 | 0.89 | 0.90 | 0.012 |
| Minimum |  | 1025 | 0.80 | 0.81 | 0.81 | 0.78 | 0.78 | 0.78 | 0.78 | 0.002 |
| Maximum |  | 2765 | 0.98 | 0.97 | 0.97 | 0.97 | 0.97 | 0.97 | 0.97 | 0.019 |

^†^ Number of individuals

^‡^ Correlation confidence interval for minimum model per trait.

**Supplemental Figure S1**. Correlation matrix between observed and adjusted phenotype values and seven different genomic selection models for spike yield 2016. Diagonal contains type of observation or model where best linear unbiased predictor (BLUP) is output from a phenotypic model and genomic estimated breeding value (GEBV) is output from a GS model, the lower triangle is a scatter plot of values, and the upper triangle corresponding the correlation value. Significance at < 0.001.

**Supplemental Figure S2**. Correlation matrix between observed phenotypes, best linear unbiased predictors (BLUPs, phenotypic adjustment), and genomic estimated breeding values (GEBVs) from genomic selection (GS) models for spike yield 2017. Diagonal contains trait values, with BLUP signifying spatial correction and GEBV indicating results from a GS model. Lower triangle is a scatter plot of variable, and the upper triangle is correlation between variables. Predictive ability for GS is reported as the GEBV and the model BLUP of the same type for example Model 3 BLUPs and Model 3 GEBV predictive ability equal 0.95. Model 1 is spatial correction with row and column AR1 and random genet term. Model 2 is spatial correction with row and column AR1 and the additive relationship matrix (**A**). Model 3 is the same as Model 2, except the **A** is replaced with the genomic realized relationship matrix, **G**RM.

**Supplemental Figure S3**. Genomic selection predictive ability for different marker numbers (Panel A) and the effect of training population size (Panel B) for spike yield, 2017. Mean value represents the results of 100 iteration at each level and intervals for standard deviation are represented. Predictive ability is reported as the correlation between genomic estimated breeding value and the phenotypic best linear unbiased predictor

.
